# Total Penetrating Keratoplasty: Indications, Therapeutic Approach, and Long-Term Follow-Up

**DOI:** 10.1155/2018/9580292

**Published:** 2018-04-19

**Authors:** Katarzyna Krysik, Ewa Wroblewska-Czajka, Anita Lyssek-Boron, Edward A. Wylegala, Dariusz Dobrowolski

**Affiliations:** ^1^Department of Ophthalmology with Pediatric Unit, St. Barbara Hospital, Trauma Center, Medykow Square 1, 41-200 Sosnowiec, Poland; ^2^Department of Ophthalmology, District Railway Hospital, Panewnicka 65 St., 40-760 Katowice, Poland; ^3^Chair and Clinical Department of Ophthalmology, School of Medicine with the Division of Dentistry in Zabrze, Medical University of Silesia in Katowice, Panewnicka 65 St., 40-760 Katowice, Poland; ^4^Hebei Provincial Eye Hospital, Xingtai, China

## Abstract

**Purpose:**

Evaluation of the indications, anatomical and functional results, and complications of total penetrating keratoplasty (TPK) in disorders involving whole cornea.

**Materials and Methods:**

We analyzed outcomes of the surgical treatment of 47 eyes of 46 patients that underwent TPK. Indications were infectious keratitis, autoimmune disease, injury of the eyeball (mainly chemical burns), and other combined disorders. The surgical technique involved dissection of affected tissues with a margin of 1.0 mm. The size of the corneal graft ranged from 10.0 to 14.0 mm. We analyzed indications, outcomes, and complications of surgery.

**Results:**

Final restoration of the ocular integrity and maintenance of the globe were achieved in 27 eyes (57%). More than one surgery was necessary in a total of 29 eyes (62%). The frequency of retransplantations did not vary significantly between the groups with different causes of corneal melting/perforation (63% of eyes with infection, 66% of eyes after trauma and 70% of eyes of patients with autoimmune disorders). Surgical treatment failed in 20 eyes (43%). Evisceration was necessary in 13 eyes (28%), phthisis occurred in 7 cases (15%).

**Conclusion:**

TPK should be considered as a last line treatment in huge corneal destruction to restore integrity of the eye globe.

## 1. Introduction

Serious corneal disorders like ulcers or perforations frequently give rise to difficult clinical situations, with rapid consecutive threatening of vision and disintegrity of the eye globe. Despite current advances in pharmacological and surgical treatment, severe corneal infections, injuries, or systemic diseases can lead to corneal perforations, which frequently require surgery “à chaud.”

Urgent reconstructive surgical interventions may be necessary to avoid consecutive endophthalmitis and the formation of the anterior and posterior synechiae and secondary glaucoma and to prevent the spread of pathogens toward the posterior pole of the eye globe or to avoid other severe complications [1–4]. The optical result of urgent surgical treatment is less important; if possible, visual restoration or rehabilitation may be accomplished in the future. Initial difficult and complicated medical status, despite a wide spectrum of pharmacological and surgical treatments, may result in fatal treatment failure with loss of the eye. The poor visual outcome of rapid corneal surgery may require further surgery [1, 4–6].

The surgical approach to retain corneal integrity may vary, depending on size, localization, depth of corneal tissue damage, and the state of other internal globe tissues. Also, coexistent or causative infection or inflammation may determine the choice and timing of surgical decisions. Additional surgical procedures like amniotic membrane transplants, conjunctival flaps, tarsorrhaphy, and botulin toxin-induced ptosis may be applied in moderate tissue damage or as the initial treatment method [5, 7, 8].

Urgent penetrating keratoplasty (PK), total penetrating keratoplasty (TPK), and corneal or corneoscleral patch graft (CSPG) combination of surgical approaches or keratoprosthesis are usually reserved for progressive, end-stage corneal pathology or last-chance medical treatment [1, 2, 9–13].

The first total penetrating keratoplasty was described in 1951 by Ramon Castroviejo as a method for replacing a large area of diseased cornea and adjacent sclera with donor tissue [14]. The primary aim of this procedure is the tectonic repair of large perforations or other extensive corneal pathologies and restoration of integrity of the globe. Because of its size, frequently involving adjacent sclera and indications for surgery (infections, corneal ulcerations, injuries, and immune disorders), total penetrating keratoplasties are associated with a high risk of complications [15–17]. The use of steroids and other immunosuppressive agents significantly reduces the rate of immunological graft rejections. Causative and complimentary intensive medication is necessary to avoid surgical treatment failure, which would lead to severe visual loss or even loss of the eye globe [5, 12, 17, 18].

The purpose of this study is to report treatment results of patients, who received a total penetrating keratoplasty for corneal diffuse destructive diseases. We review the surgical treatment, anatomical and functional results, and complications of treatment in this group of patients.

## 2. Material and Methods

We retrospectively reviewed the outcomes of the surgical treatment of 47 eyes of 46 patients that were operated on using the total penetrating keratoplasty technique. All surgeries were performed between January 1, 2010 and July 31, 2017. Patients were recruited from the Ophthalmology Department of Saint Barbara Hospital, Trauma Center, Sosnowiec, Poland, and the Chair and Clinical Department of Ophthalmology, School of Medicine with the Division of Dentistry in Zabrze, Medical University of Silesia, District Railway Hospital, Katowice, Poland. Data from the medical records included demographics, medical history, preoperative and postoperative best spectacle-corrected visual acuity (BSCVA) measured using the Snellen visual acuity (VA) chart, outcome and complications of surgery, results of accessory examinations (microbial tests), postoperative intraocular pressure, graft rejection, and other comorbidities and complications. All patients signed the informed consent form before any surgical procedure. This retrospective, observational study, according to Polish law, does not require acceptance of a local bioethical committee.

**Figure 1:**
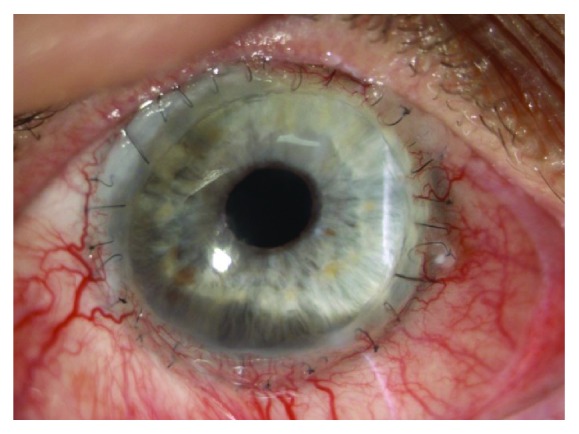
Transparent graft.

Indications for total penetrating keratoplasty were infectious keratitis, autoimmune disease, injury of the eyeball (mainly chemical burns), and other combined disorders. The main goal of the surgical treatment was the total removal of the infected or destroyed cornea and restoration of ocular integrity. After a complete ocular examination, total penetrating keratoplasty (diameter ≥ 10.0 mm) was performed (Figure 1). The surgical technique involved dissection of affected tissues with a margin of minimum 1.0 mm of nonaffected tissue. The size of the corneal graft ranged from 10.0 to 14.0 mm, depending on the extent of corneal necrosis or melting and infiltration of corneal stroma or adjacent ocular tissues. Great care was taken to avoid affecting any structures of the iridocorneal angle while preparing the recipient tissues. The oversize of the graft was 0.5 to 1.0 mm. Optimal graft size was determined by placing various-sized trephines on the recipient cornea to encompass the entire area of pathology. A 360-degree peritomy was performed at the limbus before trephination to facilitate suturing. The donor tissues (corneoscleral rings) were preserved in a cold storage media of Eusol-C solution (Alchimia, S.r.I., Ponte S. Nicolo, Italy). All surgeries were performed under general anaesthesia. To avoid consecutive glaucoma, all patients received peripheral iridectomy, unless an iridotomy or iridectomy had been performed during a preceding surgical intervention. For TPK, we used a Hanna vacuum trephine system (Moria Inc., Antony, France) or freehand trephines, if globe integrity was seriously damaged. All grafts were sutured with single 10-0 nylon sutures into the recipient sclerocorneal bed; the knots were buried. Because of the high risk of rejection in large grafts, both topical and systemic steroid medication was administrated individually, postoperatively. Also, intensive topical and general medical management for the primary ocular pathology was continued, according to its aethiology: immunosuppression, anti-inflammatory, broad-spectrum antibiotic, and antifungal or antiprotozoal therapy, respectively. Patients were also treated with cycloplegics and antiglaucomatous medications, if necessary. Because of the complex nature of underlying pathology, additional one-time or subsequent surgery was performed more than once. We followed up with all patients every two weeks for a period of three months, monthly for a minimum of six months and at differing intervals, thereafter. The mean observation time was 24 months (from 1 to 60 months).

The computer software, XLSTAT-Biomed (Addinsoft SARL, France), was used for statistical analysis and to calculate means and standard deviations. The outcome variables were not assumed to have a normal distribution, so one-way analysis of the variance was used to compare the baseline characteristics and postoperative outcomes among subgroups of causes of corneal perforation. A *p* value of <0.05 was considered statistically significant.

## 3. Results

Forty-seven eyes of forty-six patients were operated on between January 1, 2010 and July 31, 2017, using the total penetrating keratoplasty technique. This group consisted of 21 females, whose mean age was 66.13 ± 9.94 (range 39 to 80 years), and 25 males, whose mean age was 63.69 ± 14.48 (range 32 to 92 years). There was no statistically significant difference with respect to gender and age between both groups (*p* > 0.05).

All primary causes of corneal destruction and perforation requiring TPK are presented in Table 1. The main cause of this condition was infection. The most frequent infectious factors were bacterial cultures (*Pseudomonas aeruginosa*: 3 eyes (14%); *Enterococcus faecalis*: 2 eyes (9%); *Escherichia coli*: 2 eyes (9%); *Proteus mirabilis*: 3 eyes (14%); *Staphylococcus aureus*: 2 eyes (9%); and *Staphylococcus epidermidis*: 1 eye (5%)) and fungal (*Aspergillus fumigatus*: 3 eyes (14%); *Fusarium solani*: 2 eyes (9%); and *Candida albicans*: 2 eyes (9%)). Autoimmune diseases involved rheumatoid arthritis (7 eyes: 70%), ankylosing spondylitis (2 eyes: 20%), and lupus (1 eye: 10%). Eye injuries were dominated by chemical, mainly alkali, burns of the ocular surface: 8 eyes (67%). Ocular complications of Lyell's syndrome involved 2 patients (2 eyes: 100%). Neurotrophic keratopathy (loss of the neurosensory innervations of the cornea) was responsible for corneal perforation in one eye.

Final restoration of the ocular integrity and maintenance of the globe was achieved in 27 eyes (57%): 15 eyes (32%) of 15 females and 12 eyes (25%) of 12 males. More than one tectonic surgical approach, except primary total penetrating keratoplasty, was necessary in a total of 29 eyes (62%): 13 females (28%) and 16 males (34%). The frequency of repetitive tectonic corneoscleral grafting did not vary significantly between the groups of patients with different causes of corneal perforation (63% of eyes with primary infection, 66% of eyes after ocular trauma, and 70% of eyes of patients with autoimmune disorders). Characteristics of repeated tectonic surgery are reported in Table 2. Surgical treatment failed in 20 eyes (43%): 7 eyes (15%) of 7 females and 13 eyes (28%) of 13 males. Evisceration was necessary in 13 eyes (28%), and phthisis occurred in 7 eyes (15%).

Accessory simultaneous surgical procedures, not always considered before primary tectonic corneoscleral surgery, included intraocular lens explantation: 3 eyes (6%); cataract extraction or spontaneous lens extrusion at the time of surgery: 12 eyes (25%); anterior vitrectomy: 5 eyes (11%); and “open sky” pars plana vitrectomy: 4 eyes (9%).

Preoperative best spectacle-corrected visual acuity ranged from light perception (LP) to 0.05. Because the main purpose of this treatment was to retain ocular integrity, not to improve visual acuity, the final BSCVA was limited and ranged from no light perception to 0.2, after tectonic and successive (if possible) surgical and intensive additional pharmacological treatment. Visual acuity improvement was observed in 10 eyes (21%) after primary tectonic TPK and ranged from hand motion (HM) to 0.1. To achieve better final visual acuity, 11 eyes (23%) had to undergo successive, often complex, surgical treatments. Successive surgical treatment results after primary tectonic TPK are presented in Table 3.

**Figure 2:**
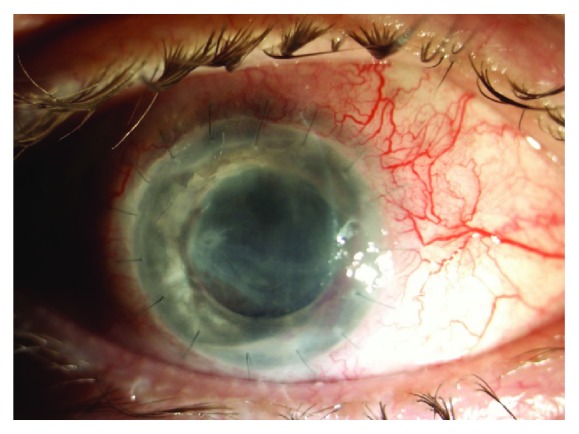
Partially transparent graft.

**Figure 3:**
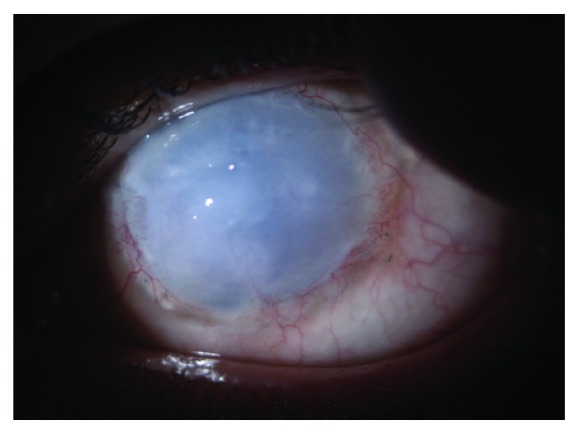
Totally opaque graft.

Graft transparency was rated during each follow-up. Final full graft transparency at last controlled visit (Figure 1) was present in 6 eyes (13%) and in 6 eyes after repetitive surgical tectonic treatment. Partial graft transparency (25–50% of corneal tissue area) (Figure 2) was achieved in 11 patients (23%) and totally opaque grafts (Figure 3) appeared in 10 patients (21%), despite repetitive surgical approaches. Postoperative complications of total penetrating keratoplasty are reported in Table 4.

The main cause of surgical treatment failure was persistent epithelial defect, observed in 34 operated eyes (72%), resulting from decreased corneal sensitivity and impaired tear production. There were 22 eyes (65%) with persistent epithelial defect refractory to medical therapy, with consecutive ulceration and perforation of the cornea requiring subsequent tectonic surgery. Repeated total penetrating keratoplasty, penetrating keratoplasty, or corneoscleral patch graft was performed on 11 eyes, including 2 eyes of 2 patients where the tectonic approach was necessary more than twice, as were the eyes of patients with Lyell's syndrome. In one eye of a patient with Lyell's syndrome, despite successive surgical approaches, eyeball atrophy occurred.

Reinfection was observed in 19 (40%) of eyes that received TPK surgery. Despite vigorous antimicrobial topical and general treatment and repeat tectonic surgery (3 penetrating keratoplasties, 2 total penetrating keratoplasties, and 1 corneoscleral patch graft), 11 (58%) of reinfected eyes developed endophthalmitis, which demanded the radical surgical approach of evisceration. Three eyes developed phthisis.

Graft melting, reported in 16 eyes (34%) and frequently preceded by loosening of the sutures and tissue necrosis resulting from infection or immunological mechanisms, was another important complication of TPK. Primary surgical treatment failed in 12 eyes (25%), and successive surgical treatment was performed: repetitive TPK in 8 eyes and CSPG in 4 eyes. Of this group, 5 eyes (31%) experienced complications from infection, 3 eyes developed phthisis, and evisceration was necessary in 2 eyes.

Early graft rejection, characterised by a whitish, sterile ring or diffuse infiltrates, was present in 5 eyes (11%) and treated for infectious corneal ulcerations. Intensive topical and systemic immunosuppressive and anti-inflammatory treatment was administered, leading to scarring and thinning of the perilimbal tissue. No urgent surgical approach was necessary.

Subsequent consecutive glaucoma or ocular hypertension occurred despite surgically performed iridectomy during tectonic TPK. Peripheral iridectomy was reported in 15 eyes (32%). To normalize refractory to medical treatment in intraocular pressure, 10 eyes (67%) required surgical intervention: 5 trabeculectomies, 3 transscleral cyclophotocoagulation, and 2 Ex-press glaucoma shunt implantations.

## 4. Discussion

The main purpose of tectonic surgery is restoration and maintenance of ocular integrity. Postoperative visual acuity and graft clarity are related to many complex immunological and physiological conditions. Anatomical integrity of the globe does not guarantee improvement of vision.

Emergency surgical strategies to close a corneal perforation depend on the size and location of the defect, aethiology, and donor corneal tissue availability. When a corneoscleral flap is not available, temporary or definitely amniotic membrane transplantation or conjunctival flap is used to close the perforation. Medical and surgical treatment of large corneal perforations, even with maximal pharmacotherapy of infection, frequently fails.

In our study, we assessed, like other authors [17, 19] that the most frequent indication for rapid tectonic treatment was infection. However, despite maximum broad-spectrum medical and surgical multistage treatment, even when repeated, the final outcome remained frequently unsatisfactory and was considered a therapeutic failure. Endophthalmitis refractory to antimicrobial and anti-inflammatory treatment required the final procedure of evisceration [3, 15].

Large grafts often are regarded as a risk factor for immunologic graft failure [20]. Our results agree with those reported by Ti et al. [15] and Jonas et al. [21]. Corneal graft melting, frequently observed in autoimmune disorders complicated by corneal perforations and usually preceded by loosening of the sutures, is also comparable in frequency, according to the foregoing author's reports.

Delayed epithelialisation or persistent epithelial defect determined a significant graft failure rate and contributed to the higher rate of ocular surface complications [3]. The final result of this complication often leads to repeated tectonic and reconstructive surgery [4, 9, 20].

In the current study, despite the uncertain and frequently inauspicious preliminary prognosis, repeated tectonic surgery and multidrug immunosuppression, overall graft survival, and final globe restoration were achieved in more than half of the eyes treated. This result is comparable with other studies [4, 18]. Groups of patients with extremely large corneoscleral grafts are usually small may be dependent on geographical and ethnic conditions (variability), and statistics may not be representative, which is also emphasized by other authors [2, 4, 15, 19, 20].

Surgical interventions with large corneal perforations frequently result in consecutive glaucoma. Our results for this complication present less frequently than in some studies [6, 15] and are compatible with the reports of Kietzmann et al. [22]. Increased intraocular pressure and secondary glaucoma after total penetrating keratoplasty and other tectonic procedures involving peripheral cornea are more frequent than after procedures involving the central part of the cornea [15, 20].

Total penetrating keratoplasty is still not a standard procedure for the treatment of corneal perforations. Penetrating keratoplasty, lamellar keratoplasties, and corneoscleral patch grafts remain the more frequently used surgical approach. Systemic immunosuppression, and often multidrug therapy, is necessary to minimize the risk of graft rejection and the necessity of repeat tectonic surgical treatment. There are no clear schemes concerning immunosuppressive treatment after CSPG [5, 12, 19, 20].

The sequential, frequently multistage, and combination surgical approach is necessary to achieve final visual acuity improvement [10, 15, 19]. We have to consider primary causative factors of corneal destruction, like severe refractory to antimicrobial treatment infections, autoimmune disorders, or severe burns of the ocular surface, as well as the final result of the tectonic primary and secondary treatment. Elective corneal surgery, like optical keratoplasty, is more secure and its final outcome is more predictable. Also, other surgical techniques, like cataract surgery with IOL implantation and secondary IOL implantation, are crucial to final visual acuity restoration [1, 4, 20, 23].

We demonstrated in this study that results of large corneoscleral grafts are unpredictable. In our opinion, this is frequently the only surgical procedure able to restore ocular integrity with simultaneous removal of infectious material, inflammatory membranes, necrotic tissues, and direct drug administration. Such an approach minimizes the risk of endophthalmitis and the spread of disease to the globe, while simultaneously increasing the probability of graft survival and potential improvement of visual acuity.

Engagement of other specialists like microbiologists and immunologists is important and therapeutically helpful. Such collaboration facilitates the optimal choice of medical treatment and reduces the rate of graft failures and the side effects of pharmacological therapy.

In conclusion, total penetrating keratoplasties, despite the high risk of intra- and postoperative severe complications, constitute a true surgical treatment alternative method for large peripheral cornea and adjacent sclera perforations. Further investigation and development of new surgical techniques are necessary to improve the final results of corneal perforation treatment. The goal should be not only restoration of ocular integrity but also improvement of visual functioning.

## Figures and Tables

**Table 1:** Causes of the corneal tissue destruction and perforation (% in brackets).

Cause of perforation	Total (*n* = 47) 100%	Female (*n* = 22) 47%	Male (*n* = 25) 53%
*Infection*	22 (46.8)	11 (23.4)	11 (23.4)
Bacterial	13 (27.6)	6 (10.6)	7 (17)
Fungal	7 (14.9)	4 (8.5)	3 (6.3)
Protozoal	2 (4.25)	1 (2.1)	1 (2.1)
*Autoimmune disease*	10 (21.3)	6 (12.8)	4 (8.5)
*Trauma*	12 (25.5)	4 (8.5)	8 (17)
Chemical (burns)	8 (17)	3 (6.3)	5 (10.6)
Penetrating	4 (8.5)	1 (2.1)	3 (6.3)
*Other*	3 (6.3)	1 (2.1)	2 (4.25)

**Table 2:** Repeated surgical tectonic treatment after primary TPK (% in brackets).

Characteristics	Total (*n* = 29) (100%)	Females (*n* = 13) (45%)	Males (*n* = 16) (55%)
Regrafts rate			
reTPK	17 (58.6)	7 (53.8)	10 (62.5)
PK	8 (27.6)	5 (38.5)	3 (18.75)
CSPG	4 (13.8)	1 (7.7)	3 (18.75)

**Table 3:** BSCVA after total penetrating keratoplasty and successive optical surgical procedures.

Characteristics	Total *n* (%) *n* = 11	Preoperative BSCVA (range)	Postoperative BSCVA (range)
*Surgical procedure*			
PK	4	LP–0.02	CF–0.1
PK + cataract surgery + PCIOL implantation	3	LP–0.01	0.05–0.2
Cataract surgery + PCIOL implantation	2	HM–0.01	0.05–0.2
Secondary PCIOL in-sulcus implantation	1	0.02	0.2
Transscleral IOL fixation	1	0.02	0.1

CF: counting fingers; HM: hand movements.

**Table 4:** Postoperative complications of total penetrating keratoplasty (% in brackets).

Primary cause of ocular disease	Infection Total *n* (%) (*n* = 22)	Autoimmune Total *n* (%) (*n* = 10)	Trauma Total *n* (%) (*n* = 12)	Other Total *n* (%) (*n* = 3)
*Complication rate*				
Persistent epithelial defect	13 (59.1)	9 (90)	9 (75)	3 (100)
Reinfection	15 (68.2)	3 (30)	1 (8.3)	0
Graft melting	8 (36.4)	7 (70)	2 (16.7)	0
Graft rejection	5 (100)	0	0	0
Glaucoma or ocular hypertension	4 (18.2)	4 (40)	6 (50)	1 (33.3)
